# Unhealthy Foods and Sugar-Sweetened Beverages Consumption Among Bangladeshi Adolescents and Their Sociodemographic Determinants: Findings From a Nationally Representative Cross-Sectional Study

**DOI:** 10.7759/cureus.41262

**Published:** 2023-07-01

**Authors:** Abu Ahmed Shamim, Md Mokbul Hossain, Fahmida Akter, Nushrat Jahan Urmy, Abu Abdullah Mohammad Hanif, Mehedi Hasan, Md Showkat Ali Khan, Mohammad Aman Ullah, Md. Mafizul I Bulbul, Malay K Mridha

**Affiliations:** 1 Center for Non-communicable Diseases and Nutrition, BRAC James P Grant School of Public Health, BRAC University, Dhaka, BGD; 2 James P Grant School of Public Health, BRAC University, Dhaka, BGD; 3 Ministry of Health and Family Welfare, Bangladesh, National Nutrition Services (NNS), Dhaka, BGD; 4 Ministry of Health and Family Welfare, Bangladesh, National Nutrition Services, Dhaka, BGD

**Keywords:** bangladesh, adolescents, snacks, sugar, sugar-sweetened beverages

## Abstract

Background: Consumption of savory crispy or fried snacks (SCFS), sugary snacks (SS), and sugar-sweetened beverages (SSB) is associated with an increased prevalence of obesity and noncommunicable diseases. We aimed to estimate the consumption of SCFS, SS, and SSB among adolescent males and females in Bangladesh and to report the factors associated with their consumption using data from a nationwide cross-sectional survey.

Methods: We interviewed 4,907 adolescent males and 4,865 females for the seven-day recall on intake of SCFS, SS, and SSB from 82 randomly selected clusters from rural, non-slum urban, and slum areas. Sociodemographic and anthropometry data were also collected.

Results: Consumption of SCFS, SS, and SSB for ≥7 times per week was reported by 11.6%, 28.9%, and 25.6% of the males and 4.9%, 24.8%, and 20.7% of the females, respectively. The weekly mean frequency of SCFS, SS, and SSB intake increased after adjustment for potential confounders among females with higher maternal education and for SCFS and SSB among males with the highest level of father’s education. Increased intake of SS and SSB for both males and females was associated with dwelling in a female-headed household. SCFS intake was higher among both males and females from the richest households. Nutritional status, both overweight and obesity, and underweight, was not associated with a more frequent intake of SCFS and SS among males and females; however, a lower frequency of intake of SSB was observed among overweight and obese males. Screen time (television viewing: none, up to 1 hour, and more than 1 hour) was not associated with consumption of SCFS and SSB among both males and females.

Conclusion: Consumption of unhealthy snacks and drinks is high among adolescents in Bangladesh and needs to be addressed through policy and program measures to abate the epidemic of obesity and associated NCD.

## Introduction

Background

Adolescence is a period of rapid growth and is the last window of opportunity for compensating for suboptimal childhood growth; current development policies call for increased investments in adolescent health, education, and well-being to ensure current and future economic and social benefits [1]. The prevalence of overweight and obesity has increased rapidly in recent years among children and adolescents across the globe, including in many low- and middle-income countries [2].

Increased consumption of unhealthy foods and beverages rich in salt, sugar, saturated fats, and trans-fats and poor in health-promoting constituents such as micronutrients and dietary fibers is a risk factor for obesity and noncommunicable diseases (NCDs) among adolescents and also in later life. In a national longitudinal study in the USA, fast food intake was found to be associated with increased weight gain during the transition from adolescence to adulthood [3]. Sugary snacks (SS) and beverages were also found to be associated with gingival inflammation and dental carriage among Indian school-going adolescents [4]. Moreover, South Asian snacks are rich in saturated and trans-fats [5]. Sweet snacks are calorie-dense and rich in sugar and fat [6]. The use of harmful and unauthorized preservatives and colors in processed foods is also widely practiced in Bangladesh and thus making them potentially harmful to health [7]. Considering the harmful effects of these unhealthy foods and drinks, the Indian Academy of Pediatrics published a guideline to discourage the consumption of these foods [8].

Purpose

As frequent intake of these calorie-dense foods and drinks in early life may contribute to a higher prevalence of obesity and NCDs, it is crucial to know their consumption and the sociodemographic factors influencing their consumption among Bangladeshi adolescents. To our knowledge, no nationally representative research reported the consumption of savory and sweet foods and beverages among adolescents in Bangladesh. The objective of this study is to elucidate the consumption of savory crispy or fried snacks (SCFS), SS, and sugar-sweetened beverages (SSB) among adolescents of Bangladesh, along with sociodemographic factors that influence their consumption using data obtained from a nationally representative cross-sectional survey. 

## Materials and methods

Study design, sampling technique, and participants

The study design and sampling procedures are described previously [9]. Data were collected as part of the Food Security and Nutrition Surveillance Project (FSNSP) being conducted in Bangladesh since 1990. This surveillance previously collected data from women and children. In the 2018-19 round of the survey, other population groups, including male and female adolescents, were included. The sample size was determined to generate national and divisional estimates for the selected indicators related to nutritional status ranging from 4% to 98% and considering the probability of type I error, α=0.05; margin of error, d=0.05 or p/2 when p ≥0.1. A design effect of 1.61 was considered to address the multistage cluster sampling technique. A required sample size of 62 adolescents from each cluster was estimated, and the national estimated sample size was 11,160 (5,580 males and 5,580 females). However, in total, 9,772 adolescents (4,907 males and 4,865 females) were interviewed from 25,371 households. 

Data were collected from 82 randomly selected clusters; 57 clusters from rural areas,15 from non-slum urban areas, and 10 from urban slum areas using the procedure described previously [9]. From each cluster, 62 female and 62 male adolescents were selected randomly from the list of all adolescents in the households, not more than one female and one male adolescent were selected from the same household.

Data collection and quality control

Five data collection teams, each comprising four to five data collectors, collected data from October 6, 2018, to October 31, 2019. A five-day-long training, including classroom sessions and field practices, was arranged for the data collectors and supervisors. The training was conducted by the investigators. The training included interview techniques using the questionnaire and also anthropometric measurement standardization training according to a previously described procedure [10]. The questionnaire was field-tested and revised. About 5% of the randomly selected interviewees were re-interviewed by the supervisors within 48 hours to ensure data quality and another 5% were directly observed by the supervisor at the time of the interview. 

Outcome variables

Three categories of unhealthy foods and drinks (savory and fried snacks, sweets, and SSB), as proposed under "optional categories" of the guide for measurement of the dietary diversity of women, are incorporated in the questionnaire [11]. Examples of such foods were also collected from published literature. SCFS included snacks that are spicy or salty (but not sweet), including commonly consumed home-prepared or commercially prepared deep-fried snacks such as pakoras (deep-fried rice/wheat batter mixed with onion, spices, vegetables), samosas and singara (deep-fried triangular or cone-shaped flour shell filled with onion, vegetables, spices, and various other ingredients), chips (deep-fried, crispy and spiced potato slice), chanachur (mixture of wheat or pulse flour chips, fried peas, peanuts, rice, spices, etc.), and fried pulses, and the foods were described in a separate paper [12]. SS include traditional milk-based sweetmeats of South Asia [13] and snacks and desserts like halwa (a sweet prepared from semolina boiled with milk/water and added sugar, butter/oil, and spices such as cinnamon and cardamom) prepared at home or sugar-containing snacks purchased from restaurants, or grocery stores, such as biscuits, cakes, chocolate, sweets/candy. SSB are defined as beverages that contain added sugars in line with the definition provided by the Centers for Disease Control, USA [14]. As part of a seven-day food frequency questionnaire, the frequency of consumption of SCFS, SS, and SSB was measured by asking questions (in Bengali) about each item with an example. For example, to collect data about the weekly frequency of SCFS, the question was, “In the last seven days, how many days did you eat savory crispy or fried snacks like chips, singara, samosa, etc.?” The interviewer recorded the number of days and, for any answer greater than or equal to one day, asked a follow-up question about the frequency of the consumption for the week preceding the interview. The same question was repeated for SS and SSB. 

Explanatory variables

For the construction of the wealth index, information about household construction and asset ownership was collected using standard questions used by Measure DHS for Demographic Health Surveys, and principal component analysis was conducted to stratify households according to relative wealth; the uppermost 20% of households was considered as the "richest" and the lowermost 20% households was considered as the poorest [15]. Individual data about age, sex, education, occupation status, food intake, and physical activity were collected from adolescents, and data about household characteristics and education, and occupation were collected from their parents or the head of the household. Information about the frequency of consumption of 10 food groups during the day preceding the interview was collected, and an individual dietary diversity score (DDS) was calculated using methods described elsewhere [16]. In the absence of a defined cutoff for adolescents, the consumption of five food groups or more was considered as an adequately diversified diet in line with previously reported Bangladeshi research to define the dietary adequacy of adolescents [17]. Adolescence is defined as boys and girls aged between 10 years to 19 years [2]. Respondents were divided into two categories by age (10 to 14 years and 15 to under 19 years). Parents were divided into five categories according to the level of educational attainments: No education (never been to school), primary incomplete, complete primary school, partial secondary school, and complete secondary school or higher.

Anthropometric measurements were taken following the FANTA anthropometry manual [10]. Height was measured using a locally made stadiometer. Weight was measured using TANITA, model UM-070 weighing scale. The body mass index (BMI) of adolescents <18 years was calculated and categorized using the WHO-recommended age and sex-specific growth charts provided in the AnthroPlus software and was categorized as thin/underweight (z-score <-2SD), normal (z-score ≥ 2SD to ≤ +1SD) and overweight and/or obese (z-score > +1SD). For the adolescents >18 years of age, adult categories were used, such as underweight (BMI < 18.5 kg/m^2^), normal weight (BMI 18.5-24.9 kg/m^2^), and overweight or obese (BMI ≥ 25.0 kg/m^2^) [18].

Statistical analysis

Because of the distinct difference in the frequency of consumption of SCFS, SS, and SSB, all the analyses were stratified by gender. A weighted analysis was carried out to show the prevalence of outcome variables and risk factor analysis. The unweighted distributions of participants by characteristics are shown in the Results section. A descriptive analysis of the sociodemographic variables (frequency distribution and percentage) of the boys and girls was performed to report their distribution. Linear regression models were run to explore the mean difference of the frequency of consumption of SCFS, SS, and SSB outcome variables by categories of exposure variables. The mean difference, 95% CI, and the p-value were shown for crude and adjusted analysis. Variables for the final multivariable regression analysis were selected from the bivariate analysis; exposure variables with a p-value ≤0.2 in the bivariate model were candidates for inclusion in the final model. The crude mean changes, adjusted mean changes, and their 95% CI were estimated. A "collinearity diagnostics" was run to check multicollinearity among the variables before performing multivariable regression. Variance inflation factors (VIF) where VIF >5 was set as a potential problem, although we did not find VIF >5, which showed an absence of multicollinearity among exposure variables. All statistical analyses were performed using the statistical software package Stata (version 17.0; StataCorp LLC, College Station, TX).

## Results

Table 1 shows the background characteristics of adolescent males and females. More than half (55.9%) of the adolescents were 10-14 years old. Less than one-fifth of the adolescents (17.5%) never went to school or did not study at school at the time of data collection, and 82.5% of adolescents were students during the data collection period. More than half (53.7%) of the adolescents’ diets were not adequately diversified. Being underweight was more common among males compared to females (males: 28.5% vs. females: 18.9%). The proportion of overweight or obesity was higher among adolescent females than males (males: 8.7% vs. females: 10.5%). About 35.6% of adolescents did not watch television at all. Overall, 37.5% of the mothers and 41.9% of the fathers of adolescents had no education. 

**Table 1:** Sociodemographic characteristics of adolescents

Characteristics	Overall (N=9,772)		Males (N=4,907)		Females (N=4,865)		p-Value
	n	%	n	%	n	%	
Age groups (years)							
10-14	5,462	55.9	2,789	56.8	2,673	54.9	0.059
15-19	4,310	44.1	2,118	43.2	2,192	45.1	
Place of residence							
Rural	6,828	69.9	3,438	70.1	3,390	69.7	0.92
Non-slum urban	1,752	17.9	873	17.8	879	18.1	
Slum	1,192	12.2	596	12.2	596	12.3	
Educational status							
Currently in school	8,065	82.5	4,112	83.8	3,953	81.3	<0.001
Never went to school or not studying at school	1,707	17.	795	16.2	912	18.8	
Religion							
Muslim	8,530	87.3	4,272	87.1	4,258	87.5	0.49
Others	1,242	12.7	635	12.9	607	12.5	
Nutritional status							
Underweight/thin	2,315	23.7	1,397	28.5	918	18.9	<0.001
Normal	6,519	66.7	3,081	62.8	3,438	70.7	
Overweight or obese	938	9.6	429	8.7	509	10.5	
Dietary diversity							
Food groups ≥5	4,521	46.3	2,354	47.9	2,167	44.5	<0.001
Food groups <5	5,251	53.7	2,553	52.0	2,698	55.5	
Television time (per day)							
None	3,479	35.6	1,678	34.2	1,801	37.0	<0.001
Up to 1 hour	3,494	35.8	1,915	39.0	1,579	32.5	
>60 minutes	2,799	28.6	1,314	26.8	1,485	30.5	
Household and parental characteristics							
Sex of the household head							
Male	6,973	71.4	3,499	71.4	3,474	71.4	0.92
Female	2,794	28.6	1,405	28.7	1,389	28.6	
Maternal education							
No education	3,665	37.5	1,835	37.4	1,830	37.6	0.88
Partial primary	1,260	12.9	633	12.9	627	12.9	
Complete primary	1,711	17.5	844	17.2	867	17.8	
Partial secondary	2,117	21.7	1,081	22.0	1,036	21.3	
Complete secondary or more	1,019	10.4	514	10.5	505	10.4	
Paternal education							
No education	4,096	41.9	2,026	41.3	2,070	42.6	0.43
Partial primary	1,041	10.7	532	10.8	509	10.5	
Complete primary	1,451	14.9	713	14.5	738	15.2	
Partial secondary	1,693	17.3	875	17.8	818	16.8	
Complete secondary or more	1,491	15.3	761	15.5	730	15.0	
Household wealth quintile							
Poorest	1,958	20.1	984	20.1	974	20.0	1.00
Poorer	1,955	20.0	981	20.0	974	20.0	
Middle	1,953	20.0	981	20.0	972	20.0	
Richer	1,958	20.1	983	20.0	975	20.1	
Richest	1,943	19.9	975	19.9	968	19.9	

Table 2 presents SCFS, SS, and SSB consumption by adolescents during the seven days preceding the interview. The weighted mean weekly frequency of SCFS consumption among males was higher than among females (males: 2.50±2.73 times vs. females: 1.58±2.11 times, p<0.001). More males (11.6%) consumed SCFS at least seven times in the previous week, and this proportion was lower among females (4.9%). Similarly, the weighted mean frequency of SS and SSB consumption during the seven days preceding the interview was higher among males (4.64±4.45 times and 3.99±6.05 times, respectively, compared to females (4.08±3.98 times and 2.90±5.08 times, respectively, p<0.001). Besides, 28.9% of males and 24.8% of females consumed SS at least seven times a week, and the corresponding figures for SSB were 25.6% of males and 20.7% of females. Overall, the weighted mean frequency of unhealthy foods and drinks (combination of SCFS, SS, and SSB) for males and females was 11.13±9.85 and 8.56±8.41, respectively, and 59.3% of males and 47.6% of females consumed these seven times or more in the week preceding the interview.

**Table 2:** Adolescent's frequency of consumption of savory crispy or fried snacks, sweet snacks, and sugar-sweetened beverages during the last seven days in Bangladesh ^a^Weighted for study design. SD=standard deviation.

Characteristics	Male	Female	Overall	Male	Female	Overall	Male	Female	Overall	Male	Female	Overall
	Never			1-3 times a week			4-6 times a week			7 or more times a week		
Savory crispy or fried snacks (SCFS)												
Mean^a^ (±SD)	Male: 2.50±2.73						Female: 1.58±2.11					p<0.001
Prevalence (%)	29.1 [24.7, 33.9]	43.6 [39.0, 48.4]	36.3 [32.3, 40.6]	43.8 [40.4, 47.2]	42.2 [39.0, 45.4]	42.9 [40.3, 45.7]	15.5 [12.3, 19.3]	9.3 [7.1, 12.2]	12.4 [9.9, 15.5]	11.6 [9.2, 14.5]	4.9 [3.9, 6.6]	8.3 [6.8 10.0]
Sweet snacks (SS)												
Mean^a^ (±SD)	Male: 4.6±4.5						Female: 4.1±3.9					p<0.001
Prevalence (%)	13.9 [10.9, 17.4]	17.0 [14.2, 20.2]	15.4 [12.8, 18.5]	36.3 [31.9, 40.9]	38.2 [34.0, 42.6]	37.3 [33.2, 41.6]	20.9 [18.3, 23.9]	19.9 [16.9, 23.4]	20. [17.9, 23.3]	28.9 [23.8, 34.5]	24.8 [19.4, 31.2]	26.9 [21.9, 32.5]
Sugar-sweetened beverages (SSB)												
Mean^a^ (±SD)	Male: 4.0±6.1						Female: 2.9±5.1					p<0.001
Prevalence (%)	43.8 [36.6, 51.2]	57.7 [47.9, 66.9]	50.7 [42.5, 58.9]	24.3 [19.9, 29.3]	18.2 [14.2, 22.9]	21.2 [17.3, 25.8]	6.3 [4.8, 8.4]	3.5 [2.3, 5.1]	4.9 [3.6, 6.6]	25.6 [16.8, 36.9]	20.7 [12.3, 32.8]	23.2 [14.6, 34.8]
Any processed food												
Mean^a^ (±SD)	Male: 11.1±9.9						Female: 8.6±8.4					p<0.001
Prevalence (%)	4.3 [2.8, 6.6]	7.7 [5.9, 10.2]	6.0 [4.4, 8.2]	10.3 [7.5, 14.0]	16.5 [13.1, 20.5]	13.4 [10.5, 16.9]	26.1 [21.8, 30.9]	28.2 [24.1, 32.7]	27.1 [23.2, 31.4]	59.3 [50.8, 67.2]	47.6 [39.4, 55.9]	53.5 [45.5, 61.3]

Table 3 displays the association between SCFS consumption during the seven days prior to the interview and sociodemographic characteristics among adolescents in Bangladesh. In the multiple linear regression model for males, after adjusting for potential confounders, the mean frequency of weekly consumption of SCFS was significantly higher among the older age group [mean difference (95% CI): 0.55 (0.20, 0.91) (p≤0.01)]; non-slum urban dwellers [mean difference (95% CI): 1.63 (0.13, 3.13) (p<0.05)]; and belonging to the richest wealth quintile [mean difference (95% CI): 0.54 (0.10, 0.99) (p<0.05)]. After adjusting for potential confounders, the mean frequency of weekly SCFS consumption was significantly lower among older females compared to the younger group [mean difference (95% CI): -0.24 (-0.45, -0.04) (p<0.05)]. The mean weekly frequency of SCFS consumption increased among females living in non-slum urban areas [mean difference [(95% CI): 0.98 (0.58, 1.38) (p<0.01)] and urban slum areas [mean difference (95% CI): 1.07 (0.28, 1.87) (p<0.001)]; and consumption of a more diverse diet [mean difference (95% CI): 0.27 (0.04, 0.49) (p<0.05)]. Household and parental characteristics such as belonging to a female-headed household were associated with a higher weekly frequency of consumption of SCFS [mean difference (95% CI): 0.19 (0.01, 0.36) (p<0.05)]. An increase in maternal education also resulted in an increase in the mean weekly frequency of SCFS consumption [mean difference [(95% CI): 0.30 (0.07, 0.54) (p<0.01)], [(95% CI): 0.31 (0.11, 0.51) (p<0.01)], and [(95% CI): 0.64 (0.09, 1.19) (p<0.05)] for mothers with complete primary education, partial secondary education, and secondary or more than that level of education, respectively. Partial primary and secondary education of fathers were also associated with a mean weekly frequency of increased consumption of SCFS (p<0.05). Females from higher quintiles of wealth were also associated with an increased mean intake of SCFS (except for one category).

**Table 3:** Sociodemographic characteristics associated with mean differences in the consumption of SCFS during the last seven days among adolescents in Bangladesh ^a^Weighted for study design. *p ≤ 0.05; **p≤ 0.01; ***p≤ 0.001. Ref.=reference category; SCFS=savory crispy or fried snacks.

	Males			Females		
Characteristics	Mean frequency of consumption (95% CI)^a^	Unadjusted mean difference (95% CI)	Adjusted mean difference (95% CI)	Mean frequency of consumption (95% CI)^a^	Unadjusted mean difference (95% CI)	Adjusted mean difference (95% CI)
Age groups (years)						
10-14	2.28 [2.03, 2.54]	Ref.	Ref.	1.74 [1.51, 1.96]	Ref.	Ref.
15-19	2.80 [2.32, 3.27]	0.51 [0.13, 0.90] **	0.55 [0.20, 0.91]**	1.37 [1.13, 1.60]	-0.37 [-0.58, -0.17]***	-0.24 [-0.45, -0.04]*
Place of residence						
Rural	2.43 [2.13, 2.74]	Ref.	Ref.	1.53 [1.32, 1.74]	Ref.	Ref.
Non-slum urban	4.32 [2.88, 5.76]	1.88 [0.41, 3.36]**	1.63 [0.13, 3.13]*	2.66 [2.36, 2.97]	1.13 [0.76, 1.50]***	0.98 [0.58, 1.38]**
Slum	3.13 [1.87, 4.39]	0.69 [-0.6, 1.99]	0.59 [-0.69, 1.87]	2.59 [1.75, 3.44]	1.06 [0.19, 1.93]**	1.07 [0.28, 1.87]***
Educational status						
Currently in school	2.47 [2.20, 2.74]	Ref.	Ref.	1.65 [1.44, 1.86]	Ref.	Ref.
Never went to school or not studying at school	2.64 [1.93, 3.34]	0.16 [-0.42, 0.75]		1.22 [1.00, 1.45]	-0.42 [-0.61, -0.24]***	-0.16 [-0.35, 0.03]
Religion						
Other than Muslim	2.56 [1.96, 3.16]	Ref.	Ref.	1.80 [1.39, 2.21]	Ref.	Ref.
Muslim	2.49 [2.17, 2.81]	-0.07 [-0.69, 0.55]		1.55 [1.34, 1.76]	-0.25 [-0.66, 0.17]	
Nutritional status						
Normal	2.57 [2.24, 2.89]	Ref.	Ref.	1.52 [1.31, 1.74]	Ref.	Ref.
Underweight/thin	2.33 [2.00, 2.67]	-0.23 [-0.50, 0.03]	-0.11 [-0.35, 0.14]	1.82 [1.51, 2.14]	0.30 [0.03, 0.57]*	0.23 [-0.02, 0.48]
Overweight/Obese	2.59 [2.08, 3.10]	0.02 [-0.37, 0.42]	-0.21 [-0.57, 0.16]	1.49 [1.26, 1.73]	-0.03 [-0.28, 0.22]	-0.15 [-0.44, 0.13]
Dietary diversity						
<5 food groups	2.43 [2.16, 2.70]	Ref.	Ref.	1.44 [1.19, 1.69]	Ref.	Ref.
≥5 food groups	2.57 [2.14, 2.99]	0.13 [-0.21, 0.48]		1.75 [1.52, 1.98]	0.31 [0.08, 0.54]**	0.27 [0.04, 0.49]*
Television viewing						
None	2.37 [2.00, 2.74]	Ref.	Ref.	1.44 [1.20, 1.67]	Ref.	Ref.
Up to 1 hour	2.50 [2.12, 2.88]	0.13 [-0.31, 0.58]	0.21 [-0.17, 0.58]	1.65 [1.39, 1.91]	0.21 [-0.05, 0.46]	0.16 [-0.06, 0.39]
>60 minutes	2.65 [2.26, 3.03]	0.28 [-0.15, 0.71]	0.29 [-0.07, 0.65]	1.66 [1.40, 1.92]	0.22 [-0.08, 0.52]	0.16 [-0.11, 0.43]
Household and parental characteristics						
Household head						
Male	2.45 [2.14, 2.77]	Ref.	Ref.	1.52 [1.32, 1.72]	Ref.	Ref.
Female	2.64 [2.25, 3.03]	0.19 [-0.14, 0.51]		1.76 [1.49, 2.03]	0.24 [0.01, 0.46]*	0.19 [0.01, 0.36]*
Maternal education						
No education	2.28 [1.86, 2.71]	Ref.	Ref.	1.26 [1.08, 1.44]	Ref.	Ref.
Partial primary	2.68 [2.30, 3.06]	0.39 [0.03, 0.76]*	0.40 [0.07, 0.74]*	1.43 [1.10, 1.76]	0.17 [-0.16, 0.49]	0.01 [-0.34, 0.33]
Complete primary	2.50 [2.12, 2.88]	0.21 [-0.13, 0.56]	0.14 [-0.17, 0.45]	1.74 [1.42, 2.06]	0.48 [0.25, 0.71]***	0.30 [0.07, 0.54]**
Partial secondary	2.59 [2.26, 2.93]	0.31 [-0.19, 0.81]	0.13 [-0.28, 0.54]	1.86 [1.61, 2.11]	0.60 [0.38, 0.81]***	0.31 [0.11, 0.51]**
Complete secondary or above	2.96 [2.5, 3.43]	0.68 [0.04, 1.32]*	0.23 [-0.29, 0.76]	2.15 [1.57, 2.72]	0.88 [0.30, 1.46]***	0.64 [0.09, 1.19]*
Paternal education						
No education	2.28 [1.91, 2.65]	Ref.	Ref.	1.30 [1.10, 1.50]	Ref.	Ref.
Partial primary	2.46 [2.08, 2.84]	0.18 [-0.20, 0.57]	0.12 [-0.21, 0.45]	1.74 [1.39, 2.08]	0.43 [0.07, 0.79]*	0.33 [0.01, 0.67]*
Complete primary	2.58 [2.20, 2.97]	0.31 [-0.04, 0.65]	0.25 [-0.11, 0.61]	1.66 [1.43, 1.88]	0.35 [0.11, 0.60]**	0.13 [-0.10, 0.36]
Partial secondary	2.73 [2.32, 3.13]	0.45 [-0.03, 0.93]	0.27 [-0.13, 0.66]	2.06 [1.70, 2.43]	0.76 [0.40, 1.13]***	0.44 [0.09, 0.79]*
Complete secondary or above	2.94 [2.48, 3.41]	0.67 [0.14, 1.19]**	0.33 [-0.13, 0.78]	1.75 [1.34, 2.17]	0.45 [0.07, 0.84]**	-0.12 [-0.42, 0.19]
Household wealth quintile						
Poorest	2.20 [1.76, 2.64]	Ref.	Ref.	1.19 [0.94, 1.45]	Ref.	Ref.
Poorer	2.18 [1.78, 2.58]	-0.02 [-0.32, 0.28]	-0.10 [-0.41, 0.20]	1.48 [1.26, 1.69]	0.28 [0.05, 0.52]*	0.23 [0.01, 0.45]*
Middle	2.47 [2.17, 2.77]	0.27 [-0.16, 0.71]	0.14 [-0.29, 0.57]	1.67 [1.42, 1.91]	0.47 [0.19, 0.75]***	0.38 [0.10, 0.66]
Richer	2.90 [2.46, 3.34]	0.70 [0.15, 1.24]*	0.43 [-0.12, 0.98]	1.73 [1.41, 2.04]	0.53 [0.12, 0.95]**	0.35 [-0.02, 0.72]**
Richest	3.09 [2.55, 3.63]	0.89 [0.29, 1.49]**	0.54 [0.10, 0.99]*	2.08 [1.60, 2.56]	0.89 [0.39, 1.39]***	0.54 [0.09, 0.98]*

The association of SS consumption during the last seven days with background characteristics among adolescents in Bangladesh is shown in Table 4. In the multiple linear regression model, after adjusting for potential confounders, the mean frequency of weekly consumption of SS was significantly lower among males residing in non-slum urban areas [mean difference (95% CI): -1.21 (-1.98, -0.4) (p<0.01)] and among males watching television for more than one hour daily [mean difference (95% CI): 0.74 (0.10, 1.39) (p<0.05)]. The mean weekly frequency of SS consumption was higher for males from female-headed households [mean difference (95% CI): 0.97 (0.21, 1.72) (p<0.05)], and males from the highest two categories of fathers’ education and one category of maternal education also had a higher mean weekly frequency of consumption. Females older in age, non-slum urban dwelling, and studying in school were associated with a lower weekly frequency of consumption of SS [mean difference (95% CI): -0.52 (-1.04, -0.05) (p<0.05); mean difference (95% CI): -0.77 (-1.42, -0.12) (p<0.05); and mean difference (95% CI): -0.74 (-1.24, -0.25), (p<0.01), respectively]. On the other hand, the mean weekly frequency of SS consumption was higher for females from female-headed households, the highest two categories of maternal education, and the highest level of wealth of the households.

**Table 4:** Sociodemographic characteristics associated with mean changes in the consumption of SS during the last seven days among adolescents in Bangladesh ^a^Weighted for study design. *p ≤ 0.05; **p≤ 0.01; ***p≤ 0.001. Ref.=reference category; SS=sweet snacks.

	Males			Females		
Characteristics	Mean frequency of consumption (95% CI)^a^	Unadjusted mean difference (95% CI)	Adjusted mean difference (95% CI)	Mean frequency of consumption (95% CI)^a^	Unadjusted mean difference (95% CI)	Adjusted mean difference (95% CI)
Age groups (years)						
10-14	4.76 [4.08, 5.45]	Ref.	Ref.	4.49 [3.79, 5.18]	Ref.	Ref.
15-19	4.48 [3.79, 5.16]	-0.29 [-0.77, 0.19]		3.53 [2.95, 4.11]	-0.95 [-1.46, -0.45]***	-0.52 [-1.04, 0.005]*
Place of residence						
Rural	4.68 [4.01, 5.34]	Ref.	Ref.	4.10 [3.48, 4.72]	Ref.	Ref.
Non-slum urban	3.75 [2.99, 4.51]	-0.93 [-1.94, 0.09]	-1.21 [-1.98, -0.44]**	3.59 [3.26, 3.92]	-0.51 [-1.21, 0.19]	-0.77 [-1.42, -0.12]*
Slum	4.50 [3.76, 5.24]	-0.18 [-1.18, 0.82]	-0.31 [-1.56, 0.94]	3.93 [3.22, 4.64]	-0.17 [-1.11, 0.78]	-0.17 [-1.26, 0.92]
Educational status						
Currently in school	4.70 [4.11, 5.29]	Ref.	Ref.	4.30 [3.65, 4.94]	Ref.	Ref.
Never went to school or not studying at school	4.36 [3.19, 5.53]	-0.34 [-1.20, 0.52]		2.92 [2.44, 3.40]	-1.37 [-1.90, -0.85]***	-0.74 [-1.24, -0.25]**
Religion						
Other than Muslim	4.91 [3.60, 6.23]	Ref.	Ref.	4.47 [3.66, 5.28]	Ref.	Ref.
Muslim	4.61 [3.95, 5.26]	-0.30 [-1.59, 0.98]		4.03 [3.41, 4.65]	-0.44 [-1.26, 0.37]	
Nutritional status						
Normal	4.63 [4.00, 5.27]	Ref.	Ref.	4.00 [3.42, 4.59]	Ref.	Ref.
Underweight/thin	4.66 [3.95, 5.37]	0.03 [-0.39, 0.45]		4.52 [3.73, 5.31]	0.52 [0.04, 1.00]*	0.29 [-0.16, 0.74]
Overweight/Obese	4.70 [3.62, 5.78]	0.07 [-0.67, 0.80]		3.68 [2.91, 4.44]	-0.32 [-0.93, 0.28]	-0.50 [-1.08, 0.09]
Dietary diversity						
<5 food groups	4.58 [3.95, 5.21]	Ref.	Ref.	4.07 [3.37, 4.78]	Ref.	Ref.
≥5 food groups	4.71 [3.91, 5.51]	0.13 [-0.48, 0.74]		4.09 [3.48, 4.69]	0.01 [-0.56, 0.58]	
Television viewing						
None	4.18 [3.69, 4.66]	Ref.	Ref.	3.79 [3.33, 4.26]	Ref.	Ref.
Up to 1 hour	4.71 [3.92, 5.49]	0.53 [-0.06, 1.12]	0.40 [-0.15, 0.94]	4.07 [3.52, 4.63]	0.28 [-0.14, 0.69]	0.04 [-0.34, 0.43]
>60 minutes	5.12 [4.23, 6.00]	0.94 [0.20, 1.68]**	0.74 [0.10, 1.39]*	4.39 [3.38, 5.41]	0.60 [-0.28, 1.48]	0.34 [-0.37, 1.04]
Household and parental characteristics						
Household head						
Male	4.38 [3.88, 4.87]	Ref.	Ref.	3.75 [3.33, 4.18]	Ref.	Ref.
Female	5.48 [4.33, 6.64]	1.11 [0.22, 1.99]*	0.97 [0.21, 1.72]*	5.09 [4.03, 6.15]	1.34 [0.53, 2.14]***	1.30 [0.58, 2.01]**
Maternal education						
No education	4.08 [3.47, 4.69]	Ref.	Ref.	3.36 [2.88, 3.83]	Ref.	Ref.
Partial primary	5.03 [4.22, 5.85]	0.96 [0.31, 1.60]**	0.84 [0.22, 1.46]**	3.80 [3.25, 4.35]	0.44 [-0.06, 0.94]	0.04 [-0.48, 0.56]
Complete primary	4.46 [3.76, 5.17]	0.39 [-0.11, 0.88]	-0.02 [-0.52, 0.49]	4.13 [3.39, 4.87]	0.77 [0.15, 1.38]*	0.46 [-0.13, 1.04]
Partial secondary	5.08 [4.30, 5.86]	1.00 [0.39, 1.62]**	0.37 [-0.18, 0.92]	4.94 [4.10, 5.77]	1.58 [0.90, 2.25]***	1.17 [0.55, 1.78]**
Complete secondary	5.99 [4.72, 7.26]	1.91 [0.85, 2.98]***	0.75 [-0.23, 1.74]	5.36 [4.21, 6.51]	2.00 [1.11, 2.90]***	1.63 [0.71, 2.55]**
Paternal education						
No education	4.07 [3.53, 4.62]	Ref.	Ref.	3.54 [3.05, 4.03]	Ref.	Ref.
Partial primary	4.35 [3.76, 4.94]	0.28 [-0.31, 0.87]	0.15 [-0.36, 0.66]	4.44 [3.77, 5.11]	0.90 [0.26, 1.54]**	0.74 [0.08, 1.39]*
Complete primary	4.97 [3.92, 6.03]	0.90 [0.16, 1.64]*	0.75 [-0.05, 1.56]	4.48 [3.47, 5.48]	0.94 [0.13, 1.75]*	0.43 [-0.30, 1.17]
Partial secondary	5.13 [4.38, 5.87]	1.05 [0.42, 1.68]***	0.78 [0.14, 1.43]*	4.65 [3.80, 5.49]	1.11 [0.45, 1.77]**	0.38 [-0.15, 0.90]
Complete secondary	6.05 [4.89, 7.21]	1.97 [1.07, 2.88]***	1.36 [0.51, 2.22]**	4.56 [3.61, 5.52]	1.03 [0.30, 1.76]**	-0.28 [-0.91, 0.36]
Household wealth quintile						
Poorest	4.22 [3.37, 5.08]	Ref.	Ref.	3.70 [3.13, 4.27]	Ref.	Ref.
Poorer	4.69 [3.88, 5.49]	0.46 [-0.13, 1.06]	0.28 [-0.24, 0.79]	3.87 [3.14, 4.61]	0.18 [-0.45, 0.80]	0.02 [-0.50, 0.55]
Lower	4.49 [3.86, 5.11]	0.27 [-0.41, 0.94]	-0.03 [-0.71, 0.65]	4.33 [3.57, 5.08]	0.63 [-0.04, 1.29]	0.35 [-0.15, 0.84]
Richer	4.60 [4.04, 5.16]	0.38 [-0.45, 1.21]	0.08 [-0.74, 0.89]	3.71 [3.23, 4.20]	0.02 [-0.49, 0.52]	-0.22 [-0.66, 0.22]
Richest	5.61 [4.60, 6.61]	1.39 [0.33, 2.45]*	0.70 [-0.14, 1.54]	5.17 [4.17, 6.17]	1.47 [0.65, 2.29]***	0.95 [0.31, 1.59]**

Table 5 displays the association of SSB consumption during the last one week with their background characteristics among adolescents in Bangladesh. After adjustment for potential confounders, the mean weekly consumption of SSB was significantly higher among males of the older age group [mean difference (95% CI): 1.21 (0.81, 1.61) (p<0.001)]. The mean weekly frequency of SSB consumption of males from non-slum urban areas, not studying at the time of the interview, and from a female-headed household were also significantly higher. The highest level of maternal education and the higher two categories of father’s education was also significantly associated with a higher mean weekly frequency of SSB consumption. On the contrary, the mean weekly frequency of SSB consumption was lower among overweight males [mean difference (95% CI): -1.11 (-1.99, -0.24) (p<0.05)].

**Table 5:** Sociodemographic characteristics associated with mean differences in the consumption of SSB during the last seven days among adolescents in Bangladesh ^§^Weighted for study design. *p≤0.05; **p≤0.01; ***p≤0. Ref.=reference category; SSB=sugar-sweetened beverages.

	Males			Females		
Characteristics	Mean frequency of consumption (95% CI)^§^	Unadjusted mean difference (95% CI)	Adjusted mean difference (95% CI)	Mean frequency of consumption (95% CI)^§^	Unadjusted mean difference (95% CI)	Adjusted mean difference (95% CI)
Age groups (years)						
10-14	3.31 [1.86, 4.76]	Ref.	Ref.	2.90 [1.46, 4.35]	Ref.	Ref.
15-19	4.94 [3.37, 6.51]	1.63 [1.07, 2.18]***	1.21 [0.81, 1.61]***	2.90 [1.53, 4.27]	0.001 [-0.43, 0.43]	
Place of residence						
Rural	3.84 [2.31, 5.36]	Ref.	Ref.	2.79 [1.34, 4.24]	Ref.	Ref.
Non-slum urban	8.41 [5.32, 11.51]	4.58 [1.13, 8.03]**	4.19 [0.003, 8.37]*	5.78 [3.25, 8.31]	2.99 [0.07, 5.91]*	2.82 [-0.57, 6.21]
Slum	4.85 [3.17, 6.52]	1.01 [-1.25, 3.28]	-0.05 [-2.82, 2.72]	4.10 [2.65, 5.54]	1.31 [-0.74, 3.36]	0.90 [-1.62, 3.42]
Educational status						
Currently in school	3.49 [2.14, 4.84]	Ref.	Ref.	2.99 [1.53, 4.45]	Ref.	Ref.
Never went to school or not studying at school	6.63 [4.37, 8.89]	3.14 [1.9, 4.39]***	2.84 [1.61, 4.08]***	2.43 [1.25, 3.6]	-0.56 [-1.24, 0.11]	-0.39 [-0.99, 0.21]
Religion						
Other than Muslim	5.64 [2.11, 9.17]	Ref.	Ref.	4.79 [1.85, 7.73]	Ref.	Ref.
Muslim	3.76 [2.36, 5.16]	-1.88 [-5.10, 1.35]		2.66 [1.35, 3.97]	-2.14 [-4.66, 0.39]	-2.18 [-4.22, -0.14]*
Nutritional status						
Normal	4.12 [2.68, 5.56]	Ref.	Ref.	2.79 [1.43, 4.14]	Ref.	Ref.
Underweight/thin	3.82 [2.14, 5.49]	-0.30 [-0.89, 0.28]	0.21 [-0.36, 0.77]	3.29 [1.54, 5.04]	0.50 [-0.19, 1.19]	0.39 [-0.20, 0.97]
Overweight/Obese	3.47 [1.94, 5.00]	-0.65 [-1.64, 0.34]	-1.11 [-1.99, -0.24]*	2.98 [1.56, 4.40]	0.19 [-0.68, 1.06]	-0.02 [-0.78, 0.73]
Dietary diversity						
<5 food groups	4.23 [2.46, 6.00]	Ref.	Ref.	3.09 [1.53, 4.65]	Ref.	Ref.
≥5 food groups	3.74 [2.45, 5.03]	-0.49 [-1.52,0.54]		2.66 [1.43,3.90]	-0.43 [-1.07, 0.21]	-0.49 [-0.97, -0.01]*
Television viewing						
None	3.62 [2.21, 5.04]	Ref.	Ref.	2.58 [1.38, 3.79]	Ref.	Ref.
Up to 1 hour	3.80 [2.28, 5.32]	0.18 [-0.94, 1.30]	0.52 [-0.29, 1.33]	2.55 [1.25, 3.85]	-0.03 [-0.86, 0.79]	-0.06 [-0.69, 0.57]
>60 minutes	4.73 [2.79, 6.68]	1.11 [-0.44, 2.65]	1.11 [-0.12, 2.34]	3.66 [1.61, 5.72]	1.08 [-0.50, 2.67]	0.93 [-0.17, 2.03]
Household and parental characteristics						
Household head						
Male	3.07 [2.04, 4.09]	Ref.	Ref.	2.04 [1.11, 2.98]	Ref.	Ref.
Female	6.88 [4.37, 9.40]	3.82 [1.96, 5.67]***	3.45 [1.85, 5.04]***	5.53 [3.12, 7.93]	3.48 [1.77, 5.19]***	3.33 [1.73, 4.93]***
Maternal education						
No education	3.70 [2.35, 5.04]	Ref.	Ref.	2.25 [1.12, 3.37]	Ref.	Ref.
Partial primary	3.48 [2.17, 4.80]	-0.21 [-0.82, 0.39]	-0.04 [-0.51, 0.43]	2.65 [1.33, 3.97]	0.40 [-0.25, 1.05]	0.001 [-0.70, 0.70]
Complete primary	3.48 [2.44, 4.51]	-0.22 [-0.87, 0.43]	-0.36 [-0.86, 0.14]	2.93 [1.55, 4.32]	0.69 [0.10, 1.27]*	0.34 [-0.18, 0.85]
Partial secondary	4.47 [2.41, 6.52]	0.77 [-0.45, 1.99]	0.52 [-0.45, 1.48]	3.54 [1.72, 5.36]	1.29 [0.38, 2.20]**	0.70 [0.001, 1.39]*
Complete secondary or above	6.26 [3.76, 8.76]	2.56 [0.86, 4.27]**	1.82 [0.59, 3.06]**	4.47 [2.44, 6.50]	2.23 [1.01, 3.44]***	1.67 [0.35, 3.00]*
Paternal education						
No education	3.30 [2.11, 4.49]	Ref.	Ref.	2.19 [1.03, 3.35]	Ref.	Ref.
Partial primary	3.71 [2.16, 5.25]	0.41 [-0.65, 1.47]	0.56 [-0.26, 1.39]	2.52 [1.26, 3.79]	0.33 [-0.25, 0.92]	0.21 [-0.32, 0.75]
Complete primary	4.03 [2.02, 6.05]	0.73 [-0.71, 2.17]	0.73 [-0.58, 2.04]	3.38 [1.54, 5.23]	1.19 [0.33, 2.06]**	0.67 [0.02, 1.33]*
Partial secondary	4.73 [2.99, 6.48]	1.43 [0.48, 2.39]**	1.42 [0.69, 2.16]***	4.25 [2.3, 6.19]	2.06 [1.03, 3.08]***	1.30 [0.59, 2.02]***
Complete secondary or above	5.84 [3.66, 8.03]	2.54 [1.14, 3.95]***	1.51 [0.60, 2.43]***	3.52 [2.15, 4.88]	1.33 [0.63, 2.03]***	0.16 [-0.61, 0.93]
Household wealth quintile						
Poorest	3.67 [1.87, 5.47]	Ref.	Ref.	2.16 [0.99, 3.32]	Ref.	Ref.
Poorer	3.95 [2.06, 5.84]	0.28 [-0.66, 1.22]	0.03 [-0.68, 0.74]	3.18 [1.27, 5.09]	1.02 [-0.13, 2.18]	0.59 [-0.36, 1.53]
Middle	3.62 [2.42, 4.82]	-0.04 [-1.05, 0.96]	-0.34 [-1.35, 0.67]	2.86 [1.31, 4.42]	0.71 [-0.16, 1.57]	-0.03 [-0.61, 0.54]
Richer	3.77 [2.58, 4.95]	0.10 [-1.27, 1.47]	-0.52 [-1.78, 0.74]	2.55 [1.50, 3.61]	0.40 [-0.45, 1.25]	-0.33 [-1.05, 0.38]
Richest	5.47 [3.59, 7.36]	1.81 [0.55, 3.06]**	0.54 [-0.32, 1.40]	4.16 [2.51, 5.81]	2.00 [0.95, 3.06]***	0.58 [-0.12, 1.27]

Girls from female households and from households with the highest two levels of maternal education had a higher weekly frequency of consumption of SSB [mean difference (95% CI): 3.33 (1.73, 4.93) (p<0.001); mean difference (95% CI): 0.70 (0.001, 1.39) (p<0.05); and mean difference (95% CI): 1.67 (0.35, 3.02) (p<0.05), respectively]. On the contrary, females from Muslim households and females who had higher DDSs had decreased weekly mean frequency of SSB intake [mean difference (95% CI): -2.18 (-4.22, -0.14) (p<0.05); mean difference (95% CI): -0.49 (-0.97, -0.01) (p<0.05)], respectively].

## Discussion

The aim of this study was to report the consumption of unhealthy foods and drinks among adolescents in Bangladesh and also to report the sociodemographic factors associated with their consumption. Using data from a nationally representative sample, we showed that SCFS, SS, and SSB consumption is high among adolescent girls and boys in Bangladesh. Only a minor fraction of the adolescents did not consume these foods and drinks within the week preceding the interview. Higher levels of maternal education, dwelling in female-headed households, and household wealth were associated with higher levels of unhealthy food and drinks consumptions. 

One of the major findings of the study was that the consumption of SCFS and SSB was higher among older adolescent boys (15-19 years) compared to younger adolescent boys. But an opposite scenario was observed among the girls, the frequency of intake of SCFS and SS was lower among older girls compared to their younger counterparts. One explanation is that the outside movement of girls is restricted after menarches, which may result in lower freedom for them to visit shops to buy such foods [19]. 

The frequency of consumption of SCFS was significantly higher among adolescent boys and girls from non-slum urban areas and for girls from slum areas compared to their counterparts from rural areas. In urban areas, more women work in formal and informal sectors. It was reported that women’s participation in economic opportunities increased the opportunity cost of their time; therefore, it may escalate the demand for nontraditional low-cost fast foods in many countries which may explain the higher intake of SCFS and SSB in the urban areas [20]. A recent analysis using nationally representative data also reported that in Bangladesh, the mean expenditure for foods consumed outside the home was higher in the urban areas for all income groups than in rural households [21]. The availability of commercially packaged products may also be another reason for higher intake.

Among adolescent boys, currently not studying in school was associated with increased consumption of SSB. No such association was observed among girls; a rather negative association was observed for SS consumption among school dropout girls. The involvement of the wage labor market is reported as a significant reason for school dropout in Bangladesh [22], so boys who are not studying might be engaged in income-earning activities and may have money and freedom to buy unhealthy drinks.

Religion was not associated with the frequency of consumption of SCFS, SS, or SSB of boys, but the frequency of intake of SSB was significantly low among girls from Muslim families. The significance of this difference is not studied in our analysis; however, programs should consider this difference for targeting interventions.

Although the nutritional status of the adolescent boys and girls was not associated with the frequency of consumption of SCFS and SS in our study, it was negatively associated with overweight and obesity among boys. An inconsistent association between unhealthy foods or drinks intake and BMI was reported earlier; for example, among Nepali adolescents, unhealthy food intake was associated with being underweight [23] but was associated with obesity among Saudi adolescents and young adults [24]. One possibility is that the obese and overweight adolescents themselves or their parents restrict the consumption of SSB, but it needs to be explored in future studies. 

The frequency of intake of a more diverse diet was not associated with the frequency of intake of SCFS, SS, and SSB of adolescent boys; for girls, it was positively associated with the frequency of consumption of SCFS but negatively with SSB in our study. It may be possible that parents do not consider these foods unhealthy and provide these to their children and adolescents, along with healthy foods. In a recent analysis, higher dietary diversity was reported to be associated with more frequent snack consumption among Bangladeshi children [25].

In our analysis, we found that watching TV was not associated with the consumption of unhealthy snacks or drinks, except that more than 1 hour of watching TV was associated with the frequency of consumption of SS among boys. One reason is that not all households own a TV, so all children are not yet exposed to TV advertisements. But with the rapid expansion of the electricity network, most Bangladeshi households are expected to own a TV in the near future. Besides, exposure to food advertisements and watching TV during a meal or snack consumption may result in consuming a considerable amount of foods and drinks [26]. It is, therefore, important to impose some regulations on the advertisement of unhealthy foods and study the effects of such restrictions [24].

A higher level of maternal education was consistently associated with a higher frequency of consumption of SCFS, SS, and SSB among girls. Among their boys, this association was less consistent, meaning increased consumption was observed among some but not for most categories of maternal education. The highest two categories of fathers’ education were associated with the frequency of consumption of SS and SSB in boys. But for girls, only a few categories of paternal education were associated with a more frequent intake of unhealthy snacks and drinks. A recent paper from Bangladesh reported that higher parental education is associated with higher dietary diversity among rural adolescents [27]. Parents provide food to their children; fathers usually buy food, while mothers usually prepare meals and offer food. It seems more educated parents not only provide a diverse diet to their children but also provide more unhealthy foods and drinks. They may also provide pocket money to their children to buy these foods and drinks [28]. It underscores the need for initiating behavior change programs for increasing awareness of the harmful effects of such foods and drinks among adolescents and their parents.

A significantly higher frequency of SCFS intake was observed among boys from the highest wealth quintile. For girls, significant associations were observed for both frequencies of SCFS and SS consumption and higher wealth status. Such findings indicated the acceptance of unhealthy foods and drinks and underscored the importance of promoting education about the potential adverse effects of these foods and drinks.

The frequency of SS and SSB intake of boys and SCFS, SS, and SSB intake of girls were higher in female-headed households. Probably women from female households had to provide more time for managing their household activities. Using nationally representative data from Bangladesh, it was reported that the proportion of household income for "foods away from the home" increases in the house where at least one female member works in the non-farm sector [29].

Programs for the reduction of unhealthy foods and drinks among adolescents should be implemented in Bangladesh, in line with the experience of other countries. One example was the introduction of a competency-based curriculum among Moroccan middle school-level adolescents that improved healthy food choices and reduced soda drink intake [30]. Imposing taxes on SSB and sugary foods was also found effective in reducing their consumption in many countries [31].

Strengths and limitations

We collected data about SCFS, SS, and SSB, but we did not collect data about individual foods within these categories, nor did we collect data about the sources of these foods and drinks; so, we are unable to say whether these are prepared at home or purchased from the market. We also did not collect data about the quantity of these foods consumed by the adolescents daily or weekly. However, the strength of the study is that we collected data from a large number of adolescents living in sites across the country; we presented the analysis by sex, which makes our findings more generalizable. We recommend future studies to identify these foods, collect data about the sources of these foods, and determine the amount of such foods and drinks consumed by the adolescents.

## Conclusions

Our analysis revealed that SCFS, SS, and SSB consumption is high among adolescents. Various sociodemographic characteristics such as parents' education, dwelling in a female-headed household, and household wealth ownership are associated with their consumption. Considering that the frequency of consumption of these foods and drinks was high among wealthy households and in households where mothers are educated, it seems that these are not considered unhealthy. However, considering the harmful health effects of SCFS, SS, and SSB, it is recommended that policymakers and program managers take initiatives to reduce the consumption of these foods and make healthy food choices available. Restricting advertisements to discourage aggressive marketing targeting adolescents and children, providing healthy meals at schools, restricting the sale of unhealthy foods at school premises, and promoting nutrition education for adolescents and their parents also warranted further attention from policymakers for improving the health and well-being of the future generation.
